# Reducing the Socio-Economic Status Achievement Gap at University by Promoting Mastery-Oriented Assessment

**DOI:** 10.1371/journal.pone.0071678

**Published:** 2013-08-08

**Authors:** Annique Smeding, Céline Darnon, Carine Souchal, Marie-Christine Toczek-Capelle, Fabrizio Butera

**Affiliations:** 1 University of Lausanne, Lausanne, Switzerland; 2 Clermont University, Université Blaise Pascal and French University Institute, Clermont-Ferrand, France; 3 Clermont University, Université Blaise Pascal, Clermont-Ferrand, France; University of Pennsylvania, United States of America

## Abstract

In spite of official intentions to reduce inequalities at University, students’ socio-economic status (SES) is still a major determinant of academic success. The literature on the dual function of University suggests that University serves not only an educational function (i.e., to improve students’ learning), but also a selection function (i.e., to compare people, and orient them towards different positions in society). Because current assessment practices focus on the selection more than on the educational function, their characteristics fit better with norms and values shared by dominant high-status groups and may favour high-SES students over low-SES students in terms of performances. A focus on the educational function (i.e., mastery goals), instead, may support low-SES students’ achievement, but empirical evidence is currently lacking. The present research set out to provide such evidence and tested, in two field studies and a randomised field experiment, the hypothesis that focusing on University’s educational function rather than on its selection function may reduce the SES achievement gap. Results showed that a focus on learning, mastery-oriented goals in the assessment process reduced the SES achievement gap at University. For the first time, empirical data support the idea that low-SES students can perform as well as high-SES students if they are led to understand assessment as part of the learning process, a way to reach mastery goals, rather than as a way to compare students to each other and select the best of them, resulting in performance goals. This research thus provides a theoretical framework to understand the differential effects of assessment on the achievement of high and low-SES students, and paves the way toward the implementation of novel, theory-driven interventions to reduce the SES-based achievement gap at University.

## Introduction

The question of whether all students have the same chances to succeed at University is still a source of concern. In democratic countries, where important steps toward the democratization of access to higher education have been made, students should indeed have equal chances to achieve. However, in the vast majority of developed and industrialized countries, students’ socio-economic status (SES) still exerts a discriminating influence on academic achievement, as low-SES students systematically underachieve when compared to high-SES students [1].

In the present research, we address the societal problem of the SES-achievement gap by focusing on a structural peculiarity of the academic system, suggesting that University serves not only an educational function (i.e., to improve students’ skills and knowledge), but also a selection function (i.e., to compare people, and orient them toward different positions in society) [2]–[4]. Usually, the competition-based selection process favors resources-endowed high-SES students [5], [6], and indeed historical analyses show that current assessment practices were originally developed with the purpose of serving high-status groups [7]. On the contrary, following the meritocratic principle, the increase in skills and knowledge is traditionally viewed as the main path to upward mobility for low-SES students, although empirical evidence is still lacking. Because current assessment practices focus usually on the selection more than on the educational function of the system, their characteristics fit better with norms and values shared by dominant high-status groups [8]. This focus on the selection function of assessment may favor high-SES students over low-SES students in terms of performances. Consequently, we suggest that the SES-based achievement gap may be due to the way achievement is assessed at University, and our aim in the present research is to test whether this gap can be reduced if assessment practices are used as a tool for education (i.e., associated to mastery goals) rather than for selection (i.e., associated to performance goals). This would support the yet untested idea that low-SES students can perform as well as high-SES students if they are led to understand assessment as part of the learning process rather than as a way to compare students to each other and select the best of them.

Research aimed at reducing the achievement gap is not novel. However, previous interventions documenting a reduction in the gap between high and low status students required either expensive special programs [9] or specific training [10]. Research on stereotype threat, instead, has demonstrated that brief interventions can reduce the threat of confirming a negative stereotype and increase low-status students’ achievement at University [11]–[13]. However, these interventions either required removing the tests’ evaluative nature, which may be unrealistic or undesirable in real-life assessment practices, or adopted an individual-level approach, which resulted in interventions designed to help targets cope with the threat individually.

Our approach focuses on structural, rather than on individual factors, namely the double function of University. We argue that the educational versus selection function of University may afford different meanings to assessment. Indeed, according to some authors [14], assessment may orient students’ attention either toward mastery goals (i.e., improving skills and learning) or toward performance goals (i.e., outperforming others and demonstrating normative success) [15], [16]. More specifically, institutional assessment practices that emphasize the selection function (e.g., comparing students based on their performance) favor a shift toward performance goals (i.e., outperforming others and demonstrating normative success), whereas practices that emphasize the educational function favor mastery goal endorsement (i.e., improving skills and learning).

The achievement goal literature [17]–[19] has long demonstrated that a strong focus on mastery goals can have a positive effect on achievement-related processes. As an example, mastery goals are linked to intrinsic interest [20], low cheating [21], high self-efficacy and cooperation [22], contrary to performance goals. Interestingly, it has sometimes been argued in this literature [23] that an educational system centered on mastery goals should favor the achievement of all students, and not only–as in traditional educational systems–the achievement of elite or privileged groups. In line with this idea, some research suggested that compared to high-status groups (men, Euro-Americans), low-status groups (women, African-Americans) suffer more from the effects of competition-based performance goals [24], [25] and benefit from mastery goals in the long run in terms of self-efficacy and learning strategies [26]. Surprisingly, no research has tested directly the idea that switching the focus from University’s competition-based performance goals (the selection function) to learning-based mastery goals (the educational function) during assessment may allow improving the academic performance of low-SES students so they could reach the same level as high-SES students. The present research provides this test.

## The Present Studies

We hypothesize that when assessment is perceived as mastery-oriented, that is, intended to promote learning as opposed to selecting students, the achievement gap between low- and high-SES students at University can be reduced. In two field studies and a randomized field experiment, we tested, for the first time, the hypothesis that when students focus on the mastery component of assessment, the socio-economic status-driven achievement gap would be reduced.

In all studies, participants’ mean grades on the French high school exit exam (Baccalauréat) were obtained from official university records and were used as covariates to control for initial academic level. Regarding SES, all students reported their mother and father’s occupations (along with age and gender) upon completion of the final exam (Studies 1 and 2) or of the statistics exam (Study 3). Based on the category of the parent with the highest SES, students were coded as either “low” or “high” SES following the coding method of the Institut National de la Statistique et des Etudes Economiques (the French equivalent of the American Census Bureau). Following this method, occupations like “labor worker” or “unemployed”, were coded as “low SES”. Occupations like “teacher” or “manager” were coded as “high SES”. If an occupation was reported for only one of the parents, then this single occupation was used to code students’ SES. The coding scheme is thoroughly described in the Additional Methodological Statistical Information S1 Supporting Information.

### Study 1

#### Method

Participants were 246 first-year psychology undergraduates at a large French university (88% female; 53% classified as low-SES; M_age_ = 19, SD = 1.21) and were part, as those in Study 2, of a larger research project on students’ motivations (see [27]). Students’ grades on two types of assessments regarding the same social psychology class were obtained from university records. These two types of assessments corresponded to a mastery-oriented continuous assessment and a final exam. Concerning the mastery-oriented continuous assessment, students were explained at the beginning of the semester that it had been designed to improve the quality of learning, help them in the learning process through regular work, and increase and consolidate their knowledge. Throughout the semester, at the end of each class, students received a list of “learning goals” for the next session. Each successive session started with the short continuous assessment of learning goals. The final exam was a traditional multiple-choice norm-based test. Students in a pilot study that used a comparable sample (N = 58; 86% female) rated the continuous assessment as more mastery-goal oriented than the final exam, F(1, 56) = 76.63, p<.001, η_p_^2^ = .58. Additional methodological information and analyses for the three main studies and the pilot study are described in the AdditionalMethodologicalStatisticalInformation S1 Supporting Information.

#### Results and Discussion

**Figure 1:**
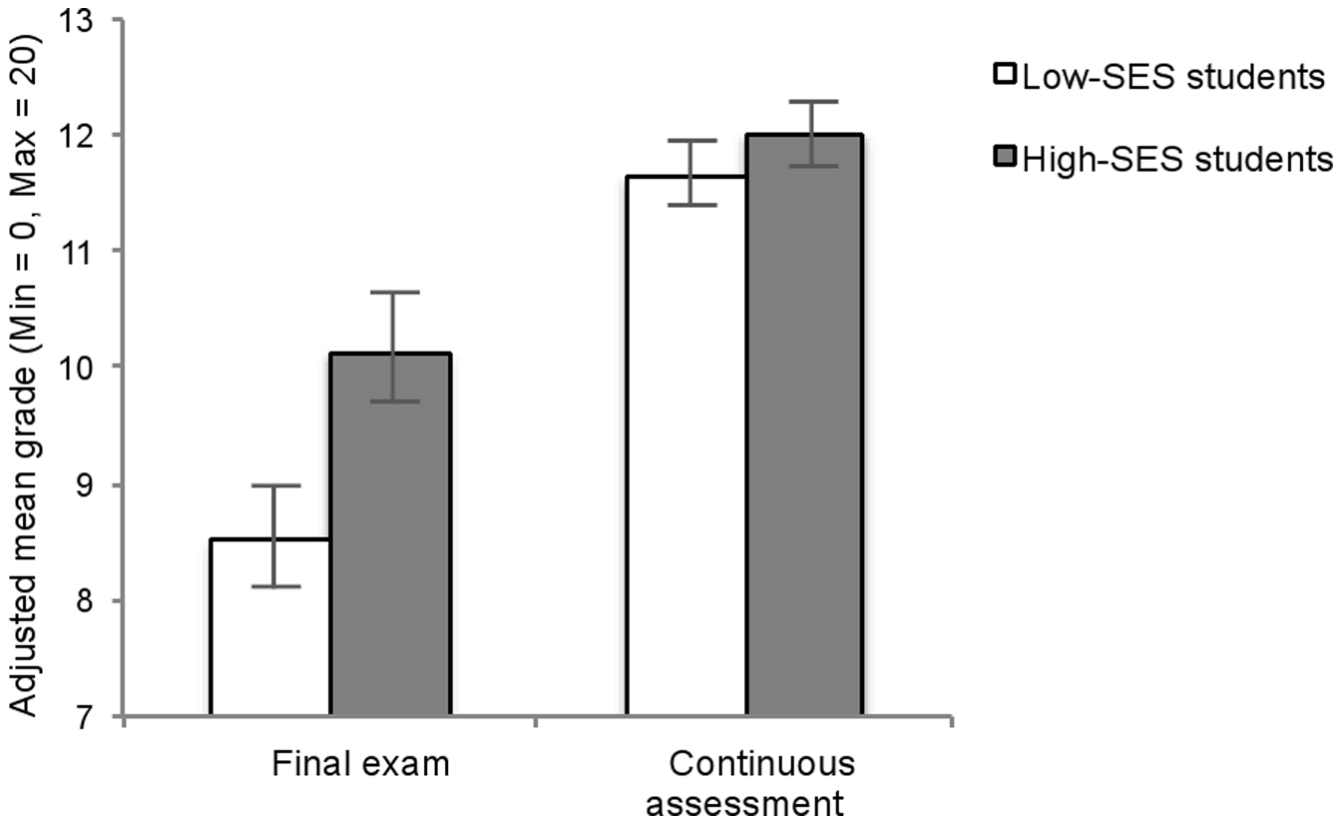
Performance pattern for study 1. Performance on the traditional multiple-choice norm-based final exam and the mastery-oriented continuous assessment in social psychology as a function of students' socio-economic status (SES) in study 1. Error bars are SEM.

We ran a mixed analysis of covariance (ANCOVA), with assessment type (mastery-oriented continuous assessment, final exam) as the within-participants variable, SES as the between-participants variable, and grade at the Baccalauréat as the covariate (all analyses are also reported without the inclusion of covariates in Supporting Information S1). Regarding the influence of SES, results revealed the classic achievement gap effect, indicating that high-SES students (M = 11.08, SE = .35) outperformed low-SES students (M = 10.09, SE = .33) regardless of assessment type, F(1, 243) = 4.16, p<.05, η_p_^2^ = .02. Moreover, grades on the mastery-oriented continuous assessment (M = 11.83, SE = .22) were higher overall compared to grades on the final exam (M = 9.33, SE = .33), F(1, 243) = 79.10, p<.001, η_p_^2^ = .25, reproducing the positive effect of formative content-based assessment [28]. Crucially, as illustrated in figure 1, the SES-by-assessment type interaction was significant, F (1, 243) = 4.83, p<.03, η_p_^2^ = .02, with high-SES students (M = 10.14, SE = .48) outperforming low-SES students (M = 8.52, SE = .45) on the final exam, F(1, 243) = 6.02, p<.02, η_p_^2^ = .02, but not on the mastery-oriented continuous assessment, p>.40. Of importance, higher performance of low-SES students on the mastery-oriented assessment as compared to the final exam condition corresponded to a shift between a failing grade and a passing grade (i.e., 10 for grades ranging from 0 to 20). This shift was not observed for high-SES students who obtained a passing grade in both assessment conditions.

Thus, the present results document that, as compared to a classical performance-based exam, an education-based assessment allows a reduction of the achievement gap between high and low SES students. One might argue, however, that the two assessments differ in more factors than just the orientation toward mastery (i.e., type of questions, delay between learning and test, test frequency, test difficulty). Therefore, in Study 2, we sought to replicate Study 1′s findings while keeping the nature of the exam constant: Only level of mastery goals varied.

### Study 2

#### Method

Participants were 233 French first-year psychology undergraduates (88% female; 53% classified as low-SES; M_age_ = 19, SD = 1.23). Their self-set mastery goals at the beginning of the semester were measured with a three-item scale (e.g., “I want to learn as much as possible from this class” [29]. Achievement corresponded to grades on the social psychology final exam only.

#### Results and discussion

**Figure 2:**
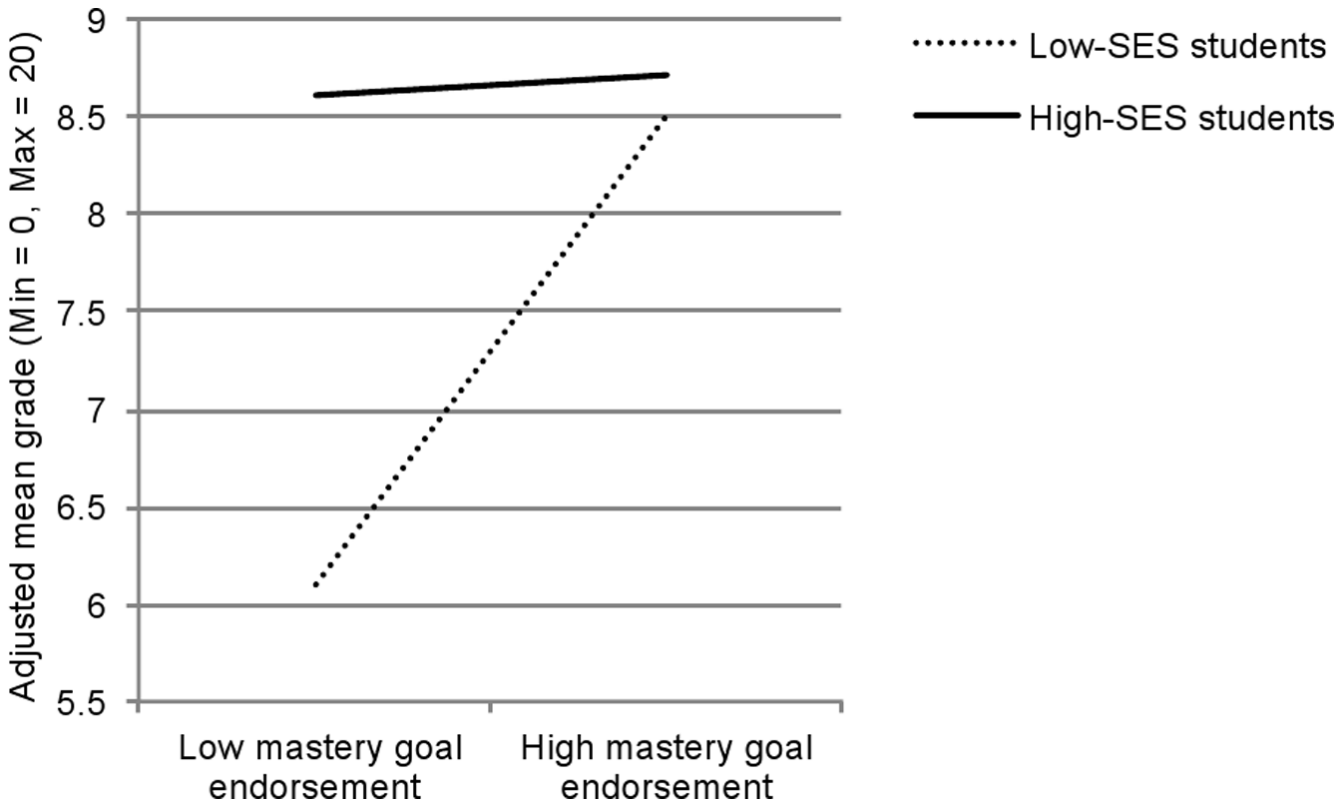
Performance pattern for study 2. Performance on the final exam as a function of students' socio-economic status (SES) and mastery goal endorsement in study 2.

We regressed social psychology final exam grades on self-set mastery goals (mean-centered), SES (low-SES students: −1, high-SES students: +1), the SES x mastery goals interaction, and Baccalauréat grades as the covariate. Results showed that high-SES students outperformed low-SES students, F(1, 228) = 5.94, p<.02, η_p_^2^ = .03, and that the higher the reported mastery goals, the better participants’ performance, F(1, 228) = 5.21, p<.03, η_p_^2^ = .02. Importantly, the SES x mastery goals interaction, F(1, 228) = 4.38, p<.04, η_p_^2^ = .02, was also significant and is depicted in figure 2. This interaction was examined by computing simple slopes for low (−1 SD) and high (+1 SD) levels of reported mastery goals. These analyses indicated that when reported level of mastery goals was low, high-SES students outperformed low-SES students, t(228) = 3.20, p<.01, but that when this level was high, there was no difference between low- and high-SES students, p>.80. Thus, even on a test that was not mastery-oriented in its form (the final exam), the SES achievement gap was reduced when students strongly endorsed mastery goals.

However, in Study 2, mastery goals were assessed using a self-reported measure, which limits the causal conclusions that can be drawn and the intervention recommendations applicable to real classroom settings. Thus, Study 3 sought to replicate our previous findings but directly manipulated, through a brief intervention, the achievement goals conveyed by the assessment while maintaining the type of assessment constant.

### Study 3

#### Method

Participants were 97 French first-year psychology undergraduates (86% female; 46% low-SES; M_age_ = 19, SD = 1.10). In this randomized field experiment, the same statistics exam was presented at the beginning of the semester as either a tool to train students (mastery-oriented assessment) or as a way to select the best of them (selection-oriented assessment) [29]. Within classes, participants were randomly assigned to the mastery-oriented assessment condition (n = 44) or to the selection-oriented assessment condition (n = 53). As some teachers were statisticians, while others were psychologists, they may have put a different emphasis on some aspects of statistics, and may have also used different illustrations in their classes (e.g., examples emphasizing psychological constructs versus more abstract constructs). We therefore controlled for teacher’s academic background in the analyses.

#### Results and discussion

**Figure 3:**
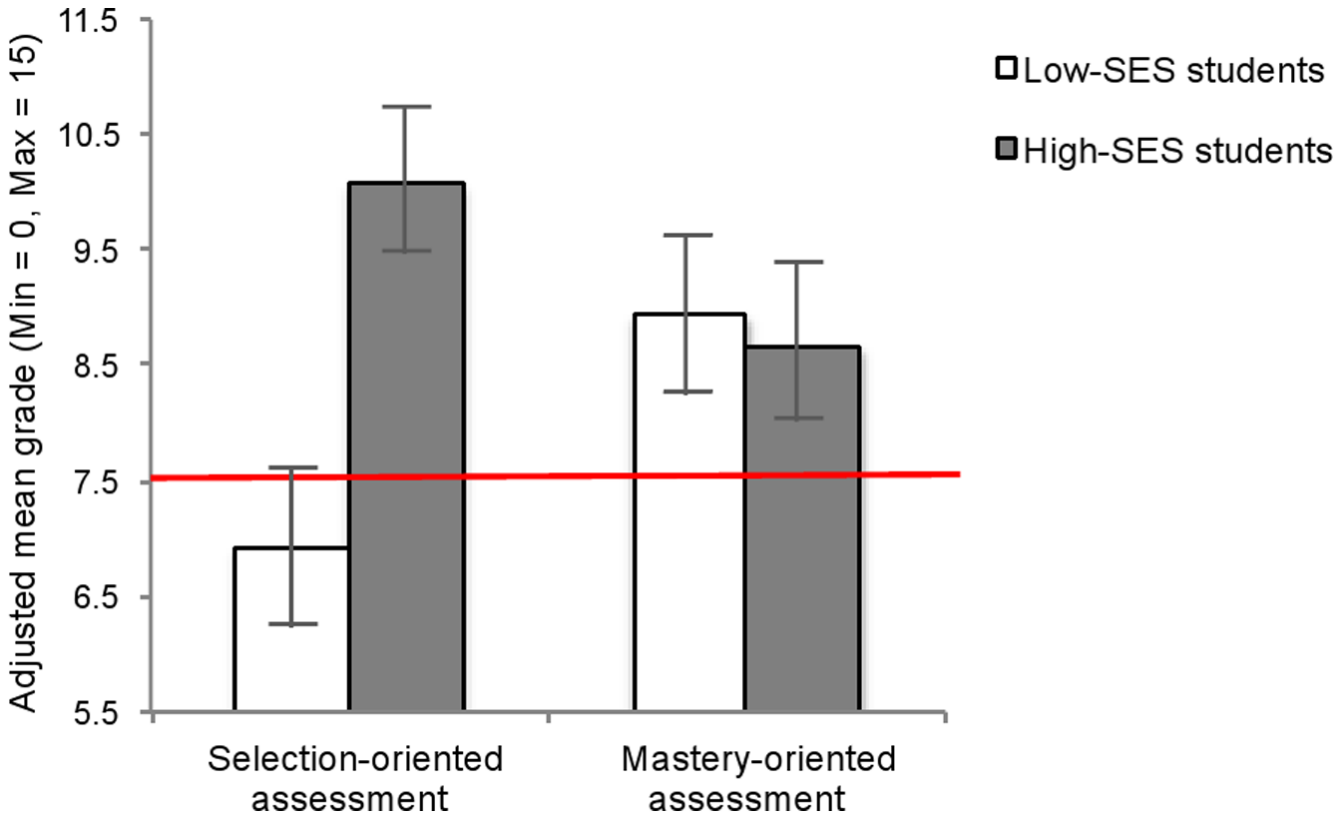
Performance pattern for study 3. Performance on the statistics exam as a function of students' socio-economic status (SES) and declared goal of assessment in Study 3. Error bars are SEM. The horizontal line represents the pass/fail grade (7.5).

A 2 (Declared goal of assessment: mastery-oriented, selection-oriented) x 2 (Socio-economic status: low, high) x 2 (Teacher’s academic background: statistics, psychology) between-participants ANCOVA was performed on statistics grades, controlling for Baccalauréat grades in mathematics (the covariate). Results showed, for the third time, but on a different subject matter, that high-SES students (M = 9.37, SE = .51) outperformed low-SES students (M = 7.93, SE = .51), F(1, 88) = 4.02, p<.05, η_p_^2^ = .04, and that, more importantly, the SES-by-declared goal of assessment interaction was significant, F(1, 88) = 5.71, p<.02, η_p_^2^ = .06: As illustrated in figure 3, high-SES students (M = 10.07, SE = .63) outperformed low-SES students (M = 6.92, SE = .70) only when the assessment was presented as a tool for selection, F(1, 88) = 11.29, p<.01, η_p_^2^ = .11, but the gap was reduced when the assessment was presented as a tool for learning, p>.80. In addition, low-SES students performed better when the assessment was presented as a tool for learning (M = 8.93, SE = .74) than when the assessment was presented as a tool for selection (M = 6.92, SE = .70), F (1, 88) = 3.96, p = .05, η_p_^2^ = .04, while this difference was not found for high-SES students, p>.16. No other main or interaction effects were significant, *ps* >.10. As in Study 1, the higher performance of low-SES students in the mastery-oriented assessment condition, as compared to the selection-oriented assessment condition, corresponded to the difference between pass and fail; but this time, we obtained this difference with a brief, randomized experimental intervention.

## General Discussion

These three studies provide convergent support for a novel approach to the SES achievement gap by focusing on the meaning of assessment practices that are used at most universities, rather than on individual factors. Using different but complementary methods, the three studies demonstrated that a focus on mastery goals in the assessment process made it possible to reduce the SES achievement gap at University. For the first time, empirical data support the idea that low-SES students can perform as well as high-SES students if they are led to understand assessment as part of the learning process rather than as a way to compare students to each other and select the best of them. Particularly the third study, which utilized an experimental design, revealed that this could be achieved with interventions that rely upon simple, albeit theory-driven instructions. Moreover, the present studies contribute to the achievement goals literature by showing that a focus on learning-based mastery goals during assessment is particularly beneficial for low-SES students. Finally, our findings may also be understood in light of the social identity threat literature [30]. Indeed, the present research suggests that some of the structural characteristics of academic functioning in terms of assessment practices may favor (i.e., selection orientation) versus reduce (i.e., mastery orientation) social identity threat for educationally-stigmatized individuals (i.e., low-SES students). Future research may investigate whether some of the mechanisms accounting for threat effects on performance (e.g., stress responses, working memory impairment; [31]) are also relevant for explaining the present findings.

Most of the time, assessment at University is associated with normative grades, ranking, and selection, but is rarely used as a genuine tool for education [32]. As our results suggest, classical performance-oriented evaluations are certainly very useful and particularly efficient in serving the selection function and maintaining the status quo [33]–[35]. However, the present research showed that mastery-oriented evaluations are far more efficient in serving the educational function and make University a place where success does not depend upon one’s social status.

### Ethics Statement

No medical or health related experimentation was performed. All studies were part of the regular assessment process at Clermont University. In Studies 1 and 2, all students completed their regular exams in exactly the same conditions and no external intervention was implemented. In Study 3, only the assigned goal varied, with all other testing conditions being strictly the same. However, since in Study 3 goals were manipulated, participants were fully debriefed and the exam did not count in their final grade. Given these regular assessment conditions, no informed consent was obtained as such a procedure is never used before taking an exam at Clermont University. All data were collected in accordance to the American Psychological Association's ethical principles and analyzed anonymously.

The Directrice de l'UFR de Psychologie, Sciences Sociales et Sciences de l'Education confirmed that informed consent from the participants was not required and that using anonymized information from University records was acceptable. The Directrice’s written approval is provided as Supporting Information S2.

Additionally, at the time the three studies were run, no approval was needed in France to conduct research on human subjects. This obligation will start on July 1st, 2014 according to law n° 2012–300 from March 5th, 2012 regarding research involving human persons (in French: LOI n° 2012–300 du 5 mars 2012 relative aux recherches impliquant la personne humaine). The Article L. 1123-6 explicitly states that the requirement to consult a Commission of Person Protection comes into effect […] at the latest July 1st, 2014 (in French: “Le a entre en vigueur […] au plus tard, le 1er juillet 2014”). The whole text, as well as the quote from the Article, can be found at http://www.legifrance.gouv.fr/affichTexte.do?cidTexte=JORFTEXT000025441587&dateTexte=&categorieLien=id (in French). Given this legislation, the present research project was not submitted to the approval of an Institutional Review Board or equivalent.

## Supporting Information

Supporting Information S1 Additional Methodological Statistical Information.
(DOCX)

Supporting Information S2 Statement Delphine Martinot Informed Consent.
(DOC)
