# Supplementary material for: Reducing the Socio-Economic Status Achievement Gap at University by Promoting Mastery-Oriented Assessment
Source: PLoS One. 2013 Aug 8;8(8):e71678. doi: 10.1371/journal.pone.0071678 (PMC3738531; doi:10.1371/journal.pone.0071678)
Supplement: Supporting Information S1 — Additional Methodological Statistical Information. (DOCX) [file pone.0071678.s001.docx]

**Supporting Information**

**Additional Methodological and Statistical Information S1**

Reducing the Socio-Economic Status Achievement Gap at University

by Promoting Mastery-Oriented Assessment

Socio-Economic Status (SES)

The Institut National de la Statistique et des Etudes Economiques (INSEE) coding method consists in categorizing individuals based on their occupational status (see http://www.insee.fr/fr/methodes/default.asp?page=nomenclatures/pcs2003/liste_n1.htm). In this official national system, these categories are 1) farm managers/owners; 2) retail traders and company heads; 3) managers and intellectual occupations; 4) intermediate occupations; 5) employees; 6) blue collar workers; 7) retired persons; 8) unemployed persons. Categories 1), 2), 3), and 4) are classified as high-SES and categories 5), 6), and 8) as low-SES. For retired persons (category 7), the occupation before retirement was used in the coding scheme if this information was available (otherwise, it was coded as missing data and the occupation of the other parent was retained). Given this coding system, and thus the nominal nature of the SES variable, treating it as a dichotomous variable was the most appropriate option. Each participant was assigned to the category of the parent with the highest SES.

**Initial Academic Level**

Participants’ grades at the Baccalauréat (the French high school exit exam) were obtained from official University records and were used as covariates to control for initial differences in academic level. In Studies 1 and 2, as the assessments concerned a social psychology course, the overall mean grade at the Baccalauréat was used (this grade corresponds to the mean of grades in various academic subjects, including French, foreign languages, mathematics and philosophy). In Study 3, as the assessment was about statistics, only the mean Baccalauréat grade in mathematics was used. The Baccalauréat grades are equivalent to SAT (verbal and/or quantitative) scores, which are often used in the United States to control for initial academic level, but which do not exist in France. For all analyses, Baccalauréat grades were mean-centered. Regarding the influence of Baccalauréat grades, results showed, in Study 1, a main effect of the covariate, F(1, 243) = 43.85, p < .001, η_p_² = .15, and an interaction effect between the covariate and assessment type, F(1, 243) = 6.78, p < .02, η_p_² = .03. In Study 2, results showed a main effect of the covariate, F(1, 228) = 31.55, p < .001, η_p_² = .12.

**Pilot study: Method and Results**

To make sure that continuous assessment would be perceived as more mastery oriented than the final exam, we conducted a pilot study on another sample of psychology undergraduates, who had experienced the two forms of assessment during their first semester. The pilot study was conducted at the beginning of the second semester. The type of assessment (continuous assessment vs. final exam) was a within-participants variable and the order of presentation was counterbalanced. For each type of assessment, participants were asked to answer the extent to which they though the final exam (vs. continuous assessment) was designed to: “help students acquire solid knowledge in psychology”; “help students learn well”; “favor a good mastery of the class”; “identify some points of the class that require to be reworked” (α = .81 for continuous assessment; α = .76 for the final exam). Participants provided ratings on a 7-point scale (1 = not at all true for me; 7= very true for me). A mean score for each assessment type was computed.

A 2 (Assessment type: continuous assessment, final exam) x 2 (Order of answer) analysis of variance (ANOVA) was performed on the data. The effect of the within-participants variable “assessment type” was significant, F (1, 56) = 76.63, p < .001, η_p_² = .58: The continuous assessment was perceived by students as more oriented toward mastery goals (M = 5.85; SD = .89) than the final exam (M = 4.11; SD = 1.38). Neither the effect of order, nor the interaction effect between order and type of assessment was significant (F < 1).

**Unreported details of Materials of Study 1**

*Mastery-Oriented Continuous Assessment*

Students had to work on the class materials at home, as well as to read the texts and to do the exercises in order to reach the learning goals before the next session. The next session then started with the short continuous assessment to evaluate the content of the learning goals. After each test, a full correction was given and questions answered. This procedure was carried out for each session but only three of these assessments were actually graded and students did not know which ones would be graded. The continuous assessment measure consisted of the mean grade obtained on the three graded tests (M = 11.83, SD = 3.70).

*Final Exam*

This was a classic final exam and contained 30 multiple-choice questions; point deduction for incorrect answers was implemented (M = 9.28, SD = 5.56).

**Unreported details of Materials of Study 2**

*Self-Set Mastery Goals*

At the beginning of the semester, participants were asked to answer the French version of [29]’s scale adapted to the social psychology class context. Participants provided ratings on a 7-point scale (1 = *not at all true for me*; 7= *very true for me*). The scale contained 3 items (“I want to learn as much as possible from this class”, “I want to understand what is taught in this class”, and “I want to master what is taught in this class”; α = .89, M = 5.59; SD = 1.16). A mean mastery-goal endorsement score was computed.

*Final exam*

This social psychology exam was similar to the final exam used in Study 1 (M = 7.98, SD = 4.58).

**Unreported details of Materials of Study 3**

*Statistics Exam and Goal of Assessment Instructions*

This exam was typical of mid-term exams in statistics (Mean grade = 8.74; SD = 2.97). It contained 15 multiple-choice and short-answer questions. For half of the participants, the exam was presented as a tool for learning (mastery goal oriented assessment), and they read the following: *“This exam has been designed to help students in their long-term learning in statistics. Indeed, this test will help you to reactivate your knowledge in statistics and to identify elements that must be improved. This exam will therefore help you to improve learning and knowledge consolidation.”* For the other half, the exam was presented as a tool for selection: *“This exam has been designed to compare students regarding their long-term learning in statistics. Indeed, this test will help you to know whether you have more knowledge in statistics than the other students and to identify differences in abilities. This exam will therefore help to make predictions regarding each student’s chances of success.”*

Upon test completion, all students were thanked, probed for suspicion, told that this exam would not be included in their final grade and fully debriefed.

**Results of the three studies without the inclusion of covariates**

**Study 1.** Results of a mixed analysis of variance (ANOVA), with SES as the between-subjects factor and type of assessment as the within-subjects factor, revealed a main effect of SES, F(1, 244) = 5.79, p < .02, η_p_² = .02, a main effect of assessment type, F(1, 244) = 76.96, p < .001, η_p_² = .24, and the SES-by-assessment type interaction, F (1, 244) = 5.75, p < .02, η_p_² = .02.

**Study 2.** Social psychology final exam grades were regressed on self-set mastery goals (mean-centered), SES (low-SES students: -1, high-SES students: +1), and the SES by mastery goals interaction. Results showed a main effect of SES, F(1, 229) = 7.19, p < .01, η_p_² = .03, a main effect of level of reported mastery goals, F(1, 229) = 5.13, p < .03, η_p_² = .02, and the SES by mastery goals interaction, F(1, 229) = 4.28, p < .04, η_p_² = .02.

**Study 3.** A 2 (Declared goal of assessment: mastery-oriented, selection-oriented) x 2 (Socio-economic status: low, high) x 2 (Teacher’s academic background: statistics, psychology) between-participants ANOVA was performed on statistics grades. Results showed a main effect of SES, F(1, 95) = 7.37, p < .01, η_p_² = .07, and the significant SES-by-declared goal of assessment interaction, F(1, 95) = 4.71, p < .03, η_p_² = .05. No other main or interaction effects were significant.
