# Supplementary material for: Reducing the Socio-Economic Status Achievement Gap at University by Promoting Mastery-Oriented Assessment
Source: PLoS One. 2013 Aug 8;8(8):e71678. doi: 10.1371/journal.pone.0071678 (PMC3738531; doi:10.1371/journal.pone.0071678)
Supplement: Supporting Information S2 — Statement Delphine Martinot Informed Consent. (DOC) [file pone.0071678.s002.doc]

| **Delphine MARTINOT**  Directrice de L'UFR de Psychologie, Sciences Sociales et Sciences de l'Education  Université Blaise Pascal  34 Avenue Carnot  63037 Clermont-Ferrand Cedex, France  Tel: 33 (0)4 73 40 64 60  Fax: 33 (0)4 73 40 64 82 | **Julia Mauchline**  PLOS ONE |
| --- | --- |

Clermont-Ferrandd, January 21 2013

Dear J. Mauchline,

I am writing to you about a research conducted by Annique Smeding, Céline Darnon and colleagues in our U.F.R..

I would like to confirm that the methodology used in the three studies matches ethical principles of psychology research and was thus accepted by our UFR.

In particular, the goal of the present research was to examine assessment conditions. To maintain high ecological validity, in such research, it is very important that students believe they are in an assessment situation when taking the test. Indeed, if students had been informed that they were actually taking part of an experiment, the situation would not have been perceived as a real assessment situation. For this reason, in such experiments, we accept that researchers do not ask participants to sign any consent form. Thus, I confirm that it was perfectly acceptable not to include informed consent from the participants in the present research.

Second, the research conducted in our UFR often requires obtaining personal data from University records (saved in paper format only). When that happens, we usually authorize researchers to access this information, as long as the data are treated anonymously. That was the case of the present research, that is why it was accepted.

For these reasons, I attest that this research was conducted with my approval.

Do not hesitate to contact me if you need any other information.

With best whishes,

La Directrice


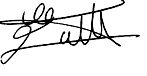


Delphine MARTINOT
